# Quantifying Age-Related Differences in Information Processing Behaviors When Viewing Prescription Drug Labels

**DOI:** 10.1371/journal.pone.0038819

**Published:** 2012-06-14

**Authors:** Raghav Prashant Sundar, Mark W. Becker, Nora M. Bello, Laura Bix

**Affiliations:** 1 School of Packaging, Michigan State University, East Lansing, Michigan, United States of America; 2 Department of Psychology, Michigan State University, East Lansing, Michigan, United States of America; 3 Department of Statistics, Kansas State University, Manhattan, Kansas, United States of America; University of Leicester, United Kingdom

## Abstract

Adverse drug events (ADEs) are a significant problem in health care. While effective warnings have the potential to reduce the prevalence of ADEs, little is known about how patients access and use prescription labeling. We investigated the effectiveness of prescription warning labels (PWLs, small, colorful stickers applied at the pharmacy) in conveying warning information to two groups of patients (young adults and those 50+). We evaluated the early stages of information processing by tracking eye movements while participants interacted with prescription vials that had PWLs affixed to them. We later tested participants’ recognition memory for the PWLs. During viewing, participants often failed to attend to the PWLs; this effect was more pronounced for older than younger participants. Older participants also performed worse on the subsequent memory test. However, when memory performance was conditionalized on whether or not the participant had fixated the PWL, these age-related differences in memory were no longer significant, suggesting that the difference in memory performance between groups was attributable to differences in attention rather than differences in memory encoding or recall. This is important because older adults are recognized to be at greater risk for ADEs. These data provide a compelling case that understanding consumers’ attentive behavior is crucial to developing an effective labeling standard for prescription drugs.

## Introduction

Successful drug interventions require the production of a safe and effective product, accurate prescribing, correct compounding and dispensing, and finally, patient compliance and adherence. Failure at any stage in the system has the potential to result in an adverse drug event (ADE), defined as “injury due to medication [1]." Adverse events in health care are increasingly recognized as an important problem, because of both health ramifications and cost [2], [3].

In 1999, the Institute of Medicine identified medication errors as a significant and preventable source of ADEs [4], [5]. It has been estimated that nearly 15 million medication errors occur annually in the US, and that a majority of them are in the outpatient setting, where it is up to the patient to use the information provided to him or her about the drug [6]. Studies have indicated that a considerable proportion of patients fail to comply with the instructions received with prescription drugs [7], [8]. Furthermore, it has been noted that more complicated drug regimens are more likely to result in an ADE. This would suggest potentially greater risk of medication errors for seniors, who are documented to have more complicated medical regimens than their younger counterparts [9].

The provision of timely information can play a part in preventing ADE’s [10], and can range from sophisticated home healthcare systems that employ technology and personnel, to simple labels affixed on prescription drug vials. Medication labels offer benefits over other approaches because they are affordable, remain with the package for the longest time and are readily accessible to the patient when needed [10], [11]. As such, proper and informative labeling is a promising and important tool in the prevention of ADEs [6], [10], [11].

**Figure 1:**
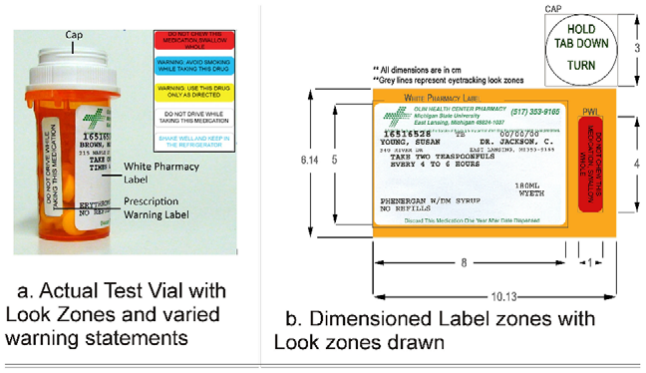
Actual vial used in this study depicting the three label zones of interest 1a - (1) cap, (2) standard white pharmacy label and (3) prescription warning label (PWL). (Inset: Five color contrasts of PWLs used in this study) 1b- Flattened, scaled drawing.

Pharmacists have attempted to capitalize on this potential by placing prescription drug warning labels (PWLs- see Figure 1) on drug vials. PWLs are small, colorful stickers that are affixed directly to the vials upon dispensing. They contain warning statements such as, “Do not consume alcohol while taking this medication" or information about routes of administration such as “For external use only." PWLs “were originally developed as a quick reminder to highlight the most important instructions for the safe and effective use of the medication." [12] Conversely, failure to heed these messages has the potential to result in an ADE.

Therefore, it is surprising that the Federal government does not regulate PWLs [12]. To date, there are no universal, federal standards regarding the method of presentation or the information conveyed by PWLs. Recognizing the need for consistent labeling of prescription drugs, the US government has recently begun to investigate approaches to standardizing the format and content of prescription drug labeling and the effect such a move would have on error rates [13], [14].

Clearly, the ability to determine the maximal potential benefits of a labeling protocol requires first identifying the optimal method of delivery for labeling. That is, a PWL may be ineffective because it is poorly designed, not because the approach of labeling medicines with PWLs is inherently ineffective.

With this in mind, there has been research investigating ways to alter PWL designs to improve their effectiveness. [5], [15] However, most of the research in this area has focused on consumers’ ability to *comprehend* warning messages, concluding that it is important to present messages in clear, simplified language and that the use of pictorial icons may be beneficial, particularly to patients with low literacy [16], [17].

While research on label comprehension is clearly an important characteristic of successful labeling, it ignores other aspects of information processing that are also critical for a label to be effective. For instance, to focus solely on comprehension presupposes that people will attend to warning labels and attempt to comprehend them. Our earlier work [18] investigating warning labels on the cartons of over the counter (OTC) drugs indicated that a significant proportion of young adults (M = 25, SD = 6.3 years of age) never *examined* two warning labels that were required by law to be prominent and conspicuous. Although OTC systems are significantly different from the prescription vials studied here (e.g. they tend to place more emphasis on marketing information), if the same holds true for the prescription warnings studied herein, it renders the question of whether or not the warning message is comprehensible, moot. That is, in the context of an information processing model [19], [20] (see Table 1), a number of serial stages of processing must occur (i.e. exposure, attention and encoding), before comprehension becomes an issue.

**Table 1:** Serial steps of a commonly recognized information processing model.

**Step 1.**	**Exposure:**	The information must be available for the patient to seek (either actively or passively)
**Step 2.**	**Attention:**	For an environmental stimulus to reach conscious awareness, it must be attended. Thus, an ideal warning will ‘stand out’ and capture the user’s attention, ensuring that it is attended even in the face of varied distractions.
**Step 3.**	**Encoding:**	The message must be extracted and encoded. The amount of cognitive resources required for the successful encoding of a stimulus is dependent upon user characteristics (i.e, the amount of cognitive capacity the individual user possesses), the information design (e.g., the legibility of the message text and complexity of the wording) and the context of interaction (e.g. well-lit, calm). Optimal informational design should reduce the required cognitive load associated with encoding the meaning of the warning, thereby, increasing the likelihood of successful encoding.
**Step 4.**	**Comprehension:**	Through encoding, an *ideal* warning will be completely converted into retrievable information in the user’s memory, enabling recall and recognition. Additionally, the intended message is comprehended.
**Step 5.**	**Compliance:**	The intended message results in the appropriate action on the part of the viewer (i.e. compliance).

Here, we focus on the early stages of the information processing model using PWLs. In particular, we investigate how attentive processes influence encoding and recognition memory of warning labels, and how factors such as the label’s color influence attention and encoding (stages 2 and 3- see Table 1). Additionally, given that older patients have been identified as at particular risk from the effects of ADEs [21]–[23], we are particularly interested in how information processing might differ between young adults and older consumers. Specifically, we assess whether the increased risk of errors among the elderly might be due primarily to deficits in encoding or memory (Table 1- Stage 3), or at least partially attributable to differences in attentive behavior (Table 1- Stage 2).

The objectives of this study were: 1) To evaluate people’s attention to PWLs using eye tracking; 2) To determine whether varying the color of PWLs impacts the probability of noticing them; 3) To investigate the relationship between attention and memory for recently presented PWLs; 4) To determine whether patterns of attention differ as a function of age and; 5) To determine the extent to which differences in attention across age groups can explain differences in recognition rates. By providing insight into the processing of information contained within PWLs, this study can inform debates about labeling designs that are most likely to impact a wide age range of consumers.

To accomplish these objectives, each subject was presented with five prescription vials, each containing a PWL in one of five color combinations (see inset Figure 1a.), in a random order of presentation. Eye tracking provided researchers with videos of a subject’s field of view with a superimposed set of crosshairs that indicated where a participant was looking. Analyzing these videos provided information on which parts of the vials were visually fixated by consumers, in what order people attended information, how many times participants returned to varied information segments and for how long. During analysis, the prescription drug vial was separated into three mutually-exclusive ‘look zones’ (see Figure 1b.) – the white pharmacy label, cap and PWL. These three zones encompassed all printed information included on the vial. While both the pharmacy label and PWLs contained important textual information about taking the medication within the vial, the cap served as a baseline condition that consisted of text not relevant to medication usage, but relevant to the operation of the vial.

In order to evaluate the attentional prioritization of label features, we modeled two dependent variables: (a) the probability of the eye ever fixating on or “hitting" a label zone, and (b) the total number of gaze shifts directed to a label zone. The probability of fixation is relatively intuitive as a dependent variable. After all, a major objective of the study was to determine whether (or not) patients looked at the PWL.

The use of the number of gaze shifts into a zone as a dependent variable may warrant further explanation. We chose this variable because it is a well-established index of the relative interest and importance of a viewing area. That is, rather than using eye movements to continually sample new information in a scene, viewers tend to repeatedly fixate on previously viewed objects when freely viewing a scene [24]. As Yarbus [25] noted, “…when changing its points of fixation, the observer’s eye repeatedly returns to the same elements… Additional time spent on perception is not used to examine the secondary elements, but to reexamine the most important elements." (p. 193) This phenomenon has been well documented in varied tasks [24] including: reading [26], [27], painting [28], problem solving [29], sorting [30], picture viewing [31] and visual search [32]. Most recently, Zelinsky et al. [24] have suggested this phenomenon to be a visual form of memory “rehearsal" needed to attenuate rapid declines in immediate memory for the important portions of a stimulus. Finally, the total number of gaze shifts to unique zones indexes how dynamic the search process is, with a high number of total gaze shifts indicative of a very dynamic process and a low number of total gaze shifts indicative of a more stationary process.

## Results

### Subjects

A total of 33 subjects were recruited for the study. Researchers failed to successfully calibrate one subject with the eye tracking equipment, so data from 32 subjects was available for analysis. Two age groups were tested, one consisting of young adults (n = 15, 7 males, 8 females, age range of 20–29 with an average age of 22.8 years) and a second, consisting of adults over the age of 50 (n = 17, 5 males, 12 females, age range of 51–77 with an average age of 62.2 years). Additional information was collected from the subjects regarding their highest level of education and number of prescription drugs taken per day; they were further characterized through the use of standardized tests that assessed their ability to see color, their level of health literacy and their visual acuity.

#### Supplementary data

Subjects from the older population reported taking an average of 2.95 prescription drugs per day (Range = 0−9), whereas the younger population reported an average of only 0.4 per day (Range = 0−2). Only one member of the older population was identified as at risk for inadequate health literacy using the REALM-R test [33], in contrast to 4 members of the younger population. All members of the younger population were found to have normal red/green color vision using Pseudo Isochromatic color plates, while two members of the older population were found to be at risk for abnormal red/green color vision.

### Attentional Prioritization - Eye Tracking Data

#### The probability of fixating a label zone (a)

Eye tracking data were first analyzed based on a binary response variable (i.e. fixated: yes, no) using a generalized linear mixed model, such that the “probability of fixating" was estimated and compared between label zones. The model included the fixed effects of population age (younger vs. older), zone (PWLs, cap and white pharmacy label) and their two-way interaction. The model also included the random effect of subject nested within population age. Explanatory variables corresponding to health literacy, number of prescription drugs per day, gender, ethnicity, age, order of presentation of vials and visual acuity of the subject were included in the statistical model during the initial stages of analysis, but were later dropped based on no evidence of improved model fit as per Bayesian Information Criteria and lack of statistical significance (P-values >0.10).

**Figure 2:**
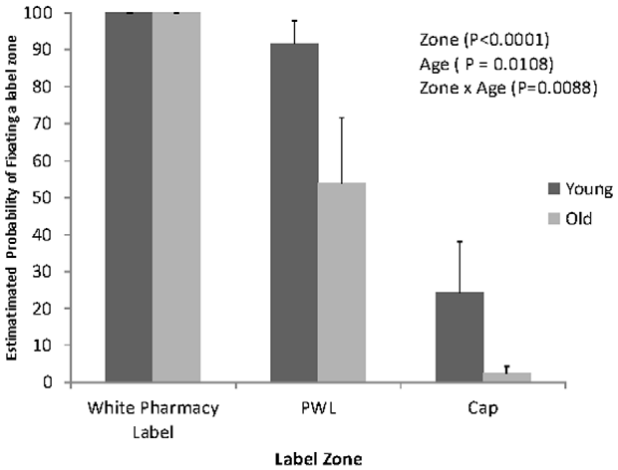
Least Square Mean Estimates (across subjects) of percentage of the probability of fixation by zone and age group. Error bars represent the between subjects standard error.

A significant age group by label zone interaction was identified on the probability of noticing a zone (P< 0.0088) (See Figure 2). More specifically, the probability of noticing a PWL was lower for the older (Estimated LSM ± SEM 54.0%±17.6%) relative to the younger population (91.8%±6.1%; P = 0.0396); yet, no evidence for age differences were apparent on the probability of noticing the white prescription label (100.0%±8.6E−7% for the older population and 100.0%±3.3E−7 for the younger population). This was also true for the probability of noticing the cap; although the relative probability of noticing the vial cap was decreased in both populations, the decrease was more pronounced in the older population (2.4%±1.95%), when compared with the younger (24.4%±13.0%; 0.0197). Within the PWL, no effect of color was evident on the probability of noticing (P = 0.9941).

#### On number of gaze shifts toward a label zone (b)

The number of gazes at a label zone was modeled using a generalized linear mixed model with a Poisson distribution. The model included the fixed effects of treatment, age group, their 2-way interaction and the effects of gender and total time spent on the vial. The effects of health literacy, number of prescription drugs taken per day, ethnicity, age, order and visual acuity were also considered but were not included in the final model due to lack of statistical significance (P-values >0.10).

This analysis revealed a significant main effect of label zone (p<0.0001), with a greater number of gazes directed at the white pharmacy label (1.35±0.11), than the PWL (0.68±0.07) (P<0.05). Fewer gazes were directed at the cap (0.20±0.04) compared to the PWL (P<0.01). This finding replicates the binary probability zone data previously discussed, and further suggests that the pharmacy label is given the highest attentional prioritization, while PWLs are given lower priority, and the cap is given the lowest priority.

**Figure 3:**
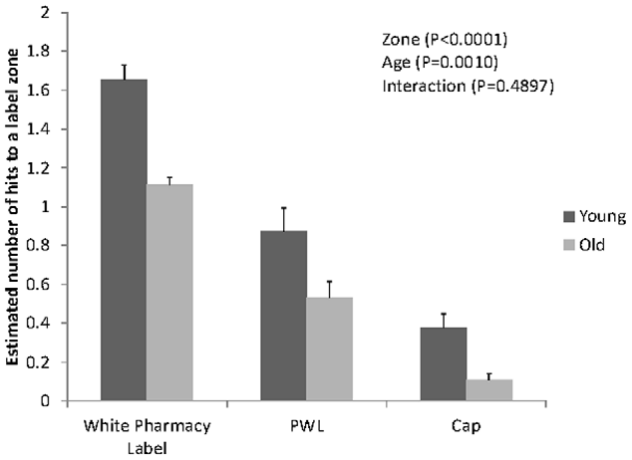
Least Square Mean Estimates of the number of gaze shifts into a label zone by age and estimated standard errors (whiskers).

A significant main effect of age group was also evident (P = 0.0010), with the younger group making more total shifts (0.85±0.09) than older participants (0.47±.06). This finding suggests that young participants implement a more dynamic attentional search comprised of more shifts of attention to different label zones. By contrast, older viewers tend to implement a more stationary process in which attention tends to shift zones infrequently (see Figure 3).

After adjusting for age group, a significant effect of gender was noted on the number of gaze shifts directed across all zones (P = 0.0099). Women were noted to have significantly more gaze shifts than their male counterparts (0.77±0.08 vs. 0.52±0.06, respectively). This suggests that women may use more dynamic search behaviors than men when seeking information from the labels of their medications. This is consistent with previous work that has indicated gender effects on hazard perception and tendencies to look for warnings [34]–[36]. There is also research that suggests that women are more likely to attend to warning information than their male counterparts [34], [37], [38]. This may be partially explained by the finding that men have a lower sense of perceived hazardousness than women towards products that are considered to be hazardous to both genders [36].

#### Recognition

After the eyetracking section of the study, subjects were presented with a printed sheet of 10 labels, five of them were the exact PWLs that they viewed on the vials and five others with different colors but the same textual messages (see the limitations section for a justification of this method). The binary response of correctly identifying the PWLs (either correctly recognized as observed or correctly rejected as not-observed during the eye tracking study), was recorded for each subject and modeled as a function of age group. In addition, the effects of health literacy, number of prescription drugs per day, ethnicity, age, gender and visual acuity were considered for model inclusion. These explanatory variables did not make significant contribution to model fit and were not included in the final model.

Recognition significantly differed between age groups (p = 0.048). The probability of correctly identifying the PWLs as seen was greater for the young (68.5%±5.05%) than the older (53.6%±4.8%) participants.

#### Fixation contingent recognition

The findings that older participants were less likely to view PWLs (see Figure 2), had fewer gaze shifts to PWLs (see Figure 3), and were less likely to correctly identify or reject the PWLs during the recognition memory test, supports the serial nature of information processing (see Table 1); i.e. that fixating on the information within the PWL is critical to its further processing. This serial process suggests that the older participants may have done worse in the recognition task, not because of problems with memory, but simply because they were less likely to attend to the PWLs during the initial viewing.

To investigate this possibility, we modeled the probability of recognition as a function of previous fixation, accounting for age groups. A generalized linear mixed model was fitted to the response “number of labels recognized" (out of the possible five presented during the eye tracking task) assuming a binomial distribution and using a logit link function. The linear predictor on the statistical model included the fixed effects of age group, fixation during eye-tracking (yes/no) and their 2-way interaction. Also included in the linear predictor was the random effect of subject nested within age group to account for subject-specific random perturbations on the binomial response explicitly.

**Figure 4:**
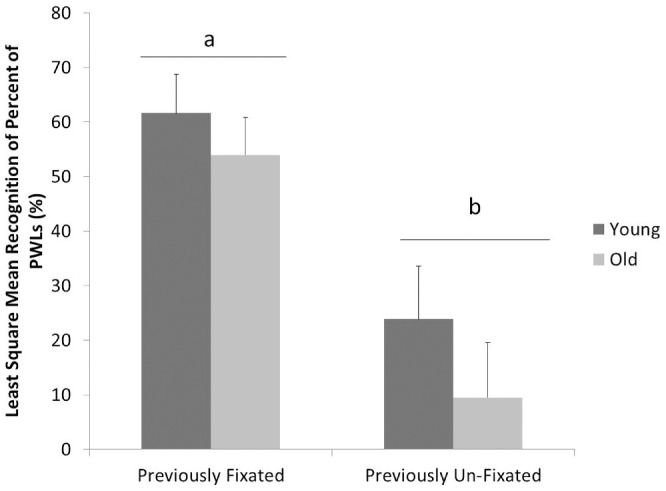
Estimated percentage of correctly recognized PWLs contingent on fixation.

This analysis revealed a significant main effect of fixation (p = 0.017), with higher recognition rates for the fixated (57.8%±7.12%) than the non-fixated objects (15.35%±6.8%) across the populations (see Figure 4). After accounting for the effect of fixation, no evidence for age differences in the probability of recognition were apparent (P = 0.17). That is, the difference in recognition between age groups may be attributable to differences in attentional allocation during the view period.

Figure 4 presents the mean estimate percentages (across subjects) of presented labels that were successfully recognized as a function of age and conditional on whether the label was fixated or not during free viewing. When subjects of either age group fixated the PWL, recognition rates were fairly high (young adults 61.7%±9.7% vs older adults 54.0%±10.1%). Similarly, when subjects from either age group failed to fixate the PWL, recognition rates were low (young 23.9%±14.5%; old 9.5%,±5.0%). Regardless of age, subjects were more likely to recognize the labels if they had fixated on them first (P = 0.0167). After accounting for fixation, no evidence for age differences in the probability of recognition were apparent (P = 0.17). Consequently, the mean difference in overall recognition rates was explained by a lower fixation rate amongst older participants.

## Discussion

We tracked eye movements while participants interacted with drug vials labeled with PWLs that are currently employed by pharmacists. We then tested participants’ recognition memory for the PWLs that they had been presented. This approach allowed us to conclude that PWLs often fail to attract attention, that this failure is more pronounced in older viewers, and this failure to attend to PWLs is associated with poor recognition memory for PWLs. These data have important implications for understanding possible shortcomings of current PWLs. They provide insight into a potential cause of age-related differences in PWL effectiveness, and suggest that designers should, at least in part, focus on attracting attention.

Presented evidence suggests that people often fail to attend to PWLs; when handed five vials in succession, only 50% of participants fixated on all five PWLs and 22% did not fixate on ANY PWLs. This lack of attention is consequential, as a failure to attend negatively impacts the ability of a PWL to be successfully encoded and remembered. Attended items were correctly recognized about 42% more often than unattended items, representing a 275% increase in memory performance relative to the base recognition rate for unattended items. As such, the findings highlight the importance of considering how a PWL impacts attention and suggests that the noted ineffectiveness of PWLs [5], [16], [17] are not limited to difficulties comprehending warning messages, but may begin earlier, with a failure to attend to the warnings (see Table 1).

Our comparison across age groups also provides some insight into the source of potential age-related differences in PWL effectiveness. Specifically, we found that only 29% of our older participants attended to all five of the PWLs, and another 29% failed to fixate any of the PWLs. For younger viewers these numbers were 73% and 13%, respectively. This dramatic difference in attention between the older and younger participants was accompanied by a lower PWL recognition rate in our older participants. More interestingly, when we compared recognition memory performance as a function of whether or not the PWL was fixated during free viewing, these age-related differences in memory performance were no longer significant. That is, both younger and older participants had similar, fairly high recognition memory for attended PWLs and similar, fairly low recognition memory for unattended PWLs. This pattern of data suggests that, at least with these brief retention intervals, the overall lower memory rates for the older participants are attributable to their failure to attend to the PWLs, rather than difficulties encoding and recalling attended labels. The implication of these age-related findings is that a label that is effective at attracting the attention of older people may more effectively convey information critical to the safe and effective use of medications in a population known to be at risk for ADEs [22], [23], [39].

Taken in total, our data suggests that a focus of PWL design should be to create PWLs that attract attention. How might one design a PWL to increase attention? Our comparison of different colored PWLs is relevant to this question. Across a variety of colors, we found that PWL color did little to increase the probability of it being noticed (P>0.70), or recognition of, the PWL. This finding was somewhat surprising to us because the presentation of a colored label should have increased the low-level visual discrepancy of the warning labels relative to the rest of the bottle. As a result, these colored labels should have been more likely to stimulate the bottom-up or saliency based attentional network [40]. The fact that increasing this bottom up signal did not significantly increase attention to the labels suggest that people’s attentive behaviors during the vial interactions were not guided by the bottom-up system, but instead were guided more by the top-down attentional system that directs attention to locations and objects that are relevant to one’s current goals [41], [42].

Consistent with this interpretation, all participants viewed the white pharmacy label, indicating that people’s expectation was that goal relevant information would be presented at that location. This interpretation suggests that the placement of the PWLs as a separate label that is spatially distinct from the white pharmacy label may actually hinder the label’s ability to garner attention (i.e. failure at step one: exposure). If the top-down attentional system is guiding attention toward the white pharmacy label, placing warning in that zone may prove to be more effective.

In conclusion, the standard types of PWLs that we tested here were not effective in capturing attention, which resulted in low recognition memory for the PWLs. In addition, these attentive deficits, and corresponding recognition deficits, were particularly acute in our older population, a population identified to be at particular risk for the ill-effects associated with ADEs [22], [23], [39]. Importantly, our analysis suggested that these age-related recognition deficits were attributable to failures to attend to the PWLs, rather than other cognitive deficits associated with aging [43]. When older participants fixated PWLs, their ability to remember the PWLs was equivalent to their younger counterparts. These data strongly suggest that attempts to improve the effectiveness of PWLs need to consider not only factors that impact later stages within the stream of information processing such as encoding and comprehension, but must also consider how the PWL impacts attention. A starting point for creating an effective PWL should be to design a label whose placement and label characteristics are likely to attract attention. Only after such a label is developed, can its impact be refined by subtle changes to wording or legibility.

## Materials and Methods

### Treatments

Five color combinations of PWL were used in this study (see Figure 1a). Four of the five color combinations (i.e. black text on blue, yellow, white and red backgrounds) were indicated by the Pharmex® (a commonly used PWL generation software) website to be the most commonly used for English and Spanish PWL’s [44]. The fifth color combination, namely blue text on a white background, was added at the request of a local pharmacist with anecdotal information that indicated this combination to be particularly problematic.

Prescription warning labels measuring 4×1 centimeters were designed using Adobe® Illustrator® CS3. Five messages were printed on the PWLs (see Figure 1a). Each message was evaluated for reading ease using the Flesch-Kincaid reading-ease score, a test that is widely used to assess readability [45]. All messages used had a reading score of exactly 66.7, indicating that they could be easily read by most 13–15 year olds.

### Vials

PWLs were attached in vertical position to 10 dram vials with a 1-clic® type closure (Figure 1a). Previous publications have indicated this vial size to be the most commonly used in the United States [46]. Along with the vertically placed PWL’s, the vials also contained a standard, white, pharmacy label which was generated by the campus pharmacy that included: dosage, drug and patient information (see Figure 1b).

All work was conducted in accordance with procedures approved by the Michigan State University SIRB under “IRB #08-246, The effect of color contrast of text on the legibility and noticeability of prescription drugs" using an informed consent process that employed both written and verbal consent.

### Eye Tracking

**Figure 5:**
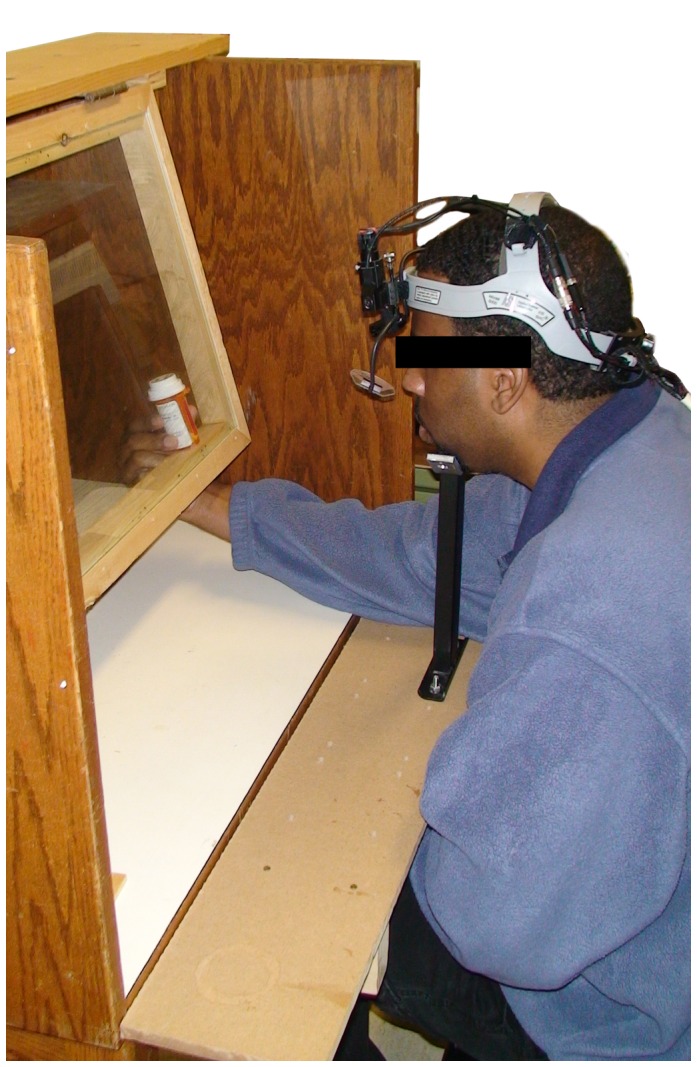
Experimental set up (chin rest, calibrated plane and the head-mounted optics of our ASL 501 eye tracking unit).

An Applied Science Laboratories (ASL - Boston, MA) Model 501® Head Mounted Optics bright-pupil eye tracker was used to track the gaze trail of subjects as they examined the prescription vials. Eye tracking data was collected in the form of video files and analyzed using Gaze Tracker® Eye Tracking analysis software. During analysis, each vial was coded into three distinct “zones:" the white pharmacy label, the PWL, and the upper surface of the white cap (see Figure 1b). Subjects were seated at a special table fixtured with a pane of a glass and a chin rest (see Figure 5). This setup allowed subjects to examine packages at a fixed distance from their eyes, minimizing parallax error and enhancing accuracy of the tracking of the gaze trail on the package surface, providing insight into the attentive behavior of the subject.

Subjects were given the following scenario, “You have just been delivered prescription medications from the pharmacy. Please do what you would normally do. Feel free to examine the vials as you please." Following this instruction, subjects were handed five pharmacy bags, one by one, each containing a single vial that included a PWL in one of the five color contrasts. The order of presentation (by PWL color contrast combination) was randomized to prevent confounding with run order. Subjects were allowed to view the vials for as long, or as little, as they wished while wearing the eye tracker, but were asked to press the package against the calibrated pane of glass while reading information on the vials. For each label zone (white pharmacy label, PWL, and cap) observed by a subject on a given vial, two dependent variables were recorded, namely the number of times the eye gaze entered the zone (i.e., “the number of gazes"), and a binary response indicating whether that zone was fixated or not. These variables were obtained by analyzing the gaze trail using the gaze tracker software and were recorded for each subject while viewing each vial.

### Recognition Memory

Once the eye tracking was complete, subjects were shown a sheet with 10 PWLs in different color contrasts; five of the color contrasts were identical to the ones they had just viewed on the vials. The other five had the same warning text as the tested PWLs but had different background colors. Subjects were asked to pick out the labels that they had just viewed on the vials. This constituted a test of recognition memory. Responses were coded in a binary fashion (correctly identified as seen or not seen previously, for a total of 10 possible correct responses).

### Supplementary Testing

Subjects were further characterized using several standardized tests. In particular, health literacy was tested using the Rapid Estimate for Adult Literacy in Medicine – Reduced (REALM-R) [33]. Also, visual acuity was determined using a Dow Corning Opthalmics Near Point Visual Acuity Card and participants’ Red/Green Color Blindness was characterized using pseudo isochromatic plates manufactured by Richmond Products® (Albuquerque, New Mexico).

### Statistical Analyses

General or generalized linear mixed models were fitted to each response of interest using the MIXED or GLIMMIX procedures of SAS, respectively (Version 9.2, SAS Institute, Cary, NC). Details on the fixed effects fitted in the linear predictor are described in the corresponding results section. The random effect of subject nested within age group was incorporated into all statistical models to explicitly account for subject-specific random perturbations on the responses and to account for lack of independence between multiple responses from a given subject. Least square mean estimates and estimated standard errors (or estimated confidence intervals) are reported. Comparisons of interest were adjusted using Tukey-Kramer’s or Bonferroni’s approach to avoid inflation of Type I error rate due to multiple comparisons.

### Limitations

It would be valuable to replicate these effects with a larger sample size that includes a greater range of ages. Although our sample size was rather limited, it granted enough statistical power to detect differences between the age groups we tested (18–29 and 50+). A larger sample size is needed to generalize the best solutions for standardization decisions.

Another potential concern is that we tested people’s memory for the specific colors of the labels rather than the specific content on the labels. While we recommend further study to probe recognition memory of label content, there were a number of reasons why we choose this approach. First, from a labeling perspective we were interested in whether certain label colors were more memorable or not. Second, within the memory literature there is a distinction between memory judgments based on familiarity and those based on recollection (for a review see Yonelinas [47]). Within this distinction, recollection is the ability to recall a specific episode or event during which information was acquired, while familiarity is the sense that one has acquired the information without specific knowledge of the circumstances under which that information was acquired [48]. In the context of PWLs, we were interested more in recollection memory than familiarity; we wanted to assess not the general knowledge that one had encountered a prescription labels that said “Do not consume alcohol while taking this medication" but specific information about the particular label/instance which had this warning. This type of recollection memory is “operationally defined as recognition accompanied by…memory for a specific feature of the study context, such as the location or color of an item." [49] (p 251). In addition this type of source memory requires deeper encoding of the stimulus [47], and is the type of memory that is most degraded in older people [50]. As such, the use of this form of memory test allowed us to assess relatively thorough encoding of the message, and probed a type of memory that older participants have the most difficulty with. Thus our finding that memory performance was no worse for older than younger subjects *provided that both had fixated the label*, is even more striking and provides strong evidence that labeling techniques which garner attention to PWLs may be beneficial to the young and old alike.
